# *N*-[2-(2-Chloro­phen­yl)-2-hy­droxy­eth­yl]propan-2-aminium benzoate

**DOI:** 10.1107/S1600536810040274

**Published:** 2010-10-23

**Authors:** Hai Feng, Ya Jian Zhou, Bin Tao Xing, Yang Guang Qi, Zheng Wu

**Affiliations:** a College of Pharmaceutical Sciences, Zhejiang University of Technology, Hangzhou 310014, People’s Republic of China; b Hangzhou Gallop Biological Products Co., Ltd, Hangzhou 310014, People’s Republic of China

## Abstract

In the title compound, C_11_H_17_ClNO^+^·C_7_H_5_O_2_^−^, obtained by the reaction of chlorprenaline {or 1-(2-chlorophenyl)-2-[(1-methylethyl)amino]ethanol} and benzoic acid, the chlorprenaline is twisted moderately [C—C—C—C torsion angle = −76.00 (17)°] compared with related compounds. The mol­ecules as usual form dimers. In the crystal structure, the two components are connected by classical O—H⋯O and N—H⋯O hydrogen bonds.

## Related literature

For related structures, see: Feld *et al.* (1981 ▶); Feng *et al.* (2010 ▶); Tang *et al.* (2009*a* ▶,*b* ▶).
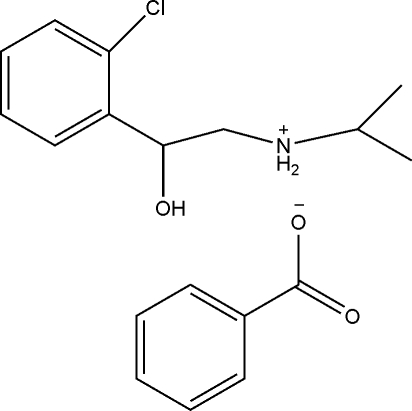


## Experimental

### 

#### Crystal data

C_11_H_17_ClNO^+^·C_7_H_5_O_2_^−^
*M* *_r_* = 335.82
Monoclinic, 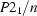

*a* = 7.8343 (3) Å
*b* = 13.1260 (5) Å
*c* = 17.7308 (7) Å
β = 94.330 (1)°
*V* = 1818.11 (12) Å^3^
*Z* = 4
Mo *K*α radiation
μ = 0.22 mm^−1^
*T* = 296 K
0.53 × 0.48 × 0.46 mm

#### Data collection

Rigaku R-AXIS RAPID/ZJUG diffractometer
Absorption correction: multi-scan (*ABSCOR*; Higashi, 1995 ▶) *T*_min_ = 0.871, *T*_max_ = 0.904
17400 measured reflections
4123 independent reflections
3186 reflections with *I* > 2σ(*I*)
*R*_int_ = 0.023

#### Refinement

*R*[*F* ^2^ > 2σ(*F* ^2^)] = 0.042
*wR*(*F* ^2^) = 0.110
*S* = 1.00
4123 reflections
210 parameters
H-atom parameters constrained
Δρ_max_ = 0.31 e Å^−3^
Δρ_min_ = −0.32 e Å^−3^

### 

Data collection: *PROCESS-AUTO* (Rigaku/MSC, 2006 ▶); cell refinement: *PROCESS-AUTO*; data reduction: *CrystalStructure* (Rigaku/MSC, 2007 ▶); program(s) used to solve structure: *SHELXS97* (Sheldrick, 2008 ▶); program(s) used to refine structure: *SHELXL97* (Sheldrick, 2008 ▶); molecular graphics: *ORTEP-3* (Farrugia, 1997 ▶); software used to prepare material for publication: *WinGX* (Farrugia, 1999 ▶).

## Supplementary Material

Crystal structure: contains datablocks global, I. DOI: 10.1107/S1600536810040274/rk2237sup1.cif

Structure factors: contains datablocks I. DOI: 10.1107/S1600536810040274/rk2237Isup2.hkl

Additional supplementary materials:  crystallographic information; 3D view; checkCIF report

## Figures and Tables

**Table 1:** Hydrogen-bond geometry (Å, °)

*D*—H⋯*A*	*D*—H	H⋯*A*	*D*⋯*A*	*D*—H⋯*A*
N1—H1*A*⋯O2	0.90	1.85	2.7231 (15)	162
O1—H1⋯O3	0.82	1.93	2.7219 (15)	162
N1—H1*B*⋯O3^i^	0.90	1.88	2.7710 (15)	169

Symmetry code: (i) 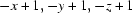.

**Fig. 1.**
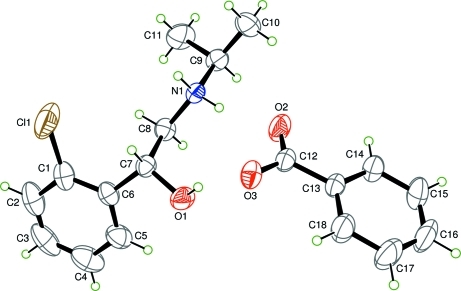
The asymmetric unit of I with atom numbering scheme. Displacement ellipsoids are drawn at 50% probability level. H atoms are presented as a small spheres of arbitrary radius.

**Fig. 2.**
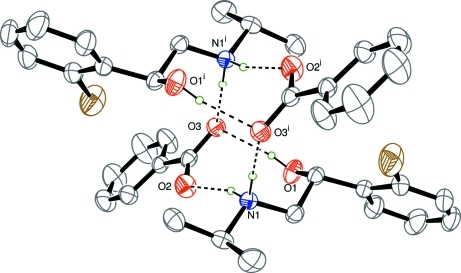
The hydrogen bonds (dashed lines) system in I.

## supplementary crystallographic information

### Comment

A recent study reports the structure of bis{*N*-[2-(2-chlorophenyl)-2-hydroxyethyl]propan-2-aminium} oxalate (Tang *et al.*, 2009*b*), which was synthesized by oxalic acid and chlorprenaline (Tang *et al.*, 2009*a*). Here using benzoic acid instead of oxalic acid and following a similar synthetic procedure yields the title compound, I.

In I (Fig. 1), the chlorprenaline molecule and the benzoic acid molecule are linked to each other by the N1—H1A···O2 hydogen bond (2.7231 (15)Å) and the O1—H1···O3 hydogen bond (2.7219 (15)Å) (Fig. 2 & Table 1). The chlorprenaline in I are twisted moderately as compared with those of other compounds. The C12—O2 distance of 1.2456 (18)Å is much shorter than the similar distance of 1.2675 (15)Å (Feld *et al.*, 1981). The C1—C6—C7—C8 torsion angle of -76.00 (17)° (104.0 (17)°) is larger than the value of the similar torsion angle of 91.9 (2)° (Tang *et al.*, 2009*a*). The C9—N1 distance of 1.5096 (17)Å is longer than the value of the similar bond distance of 1.473 (4)Å (Tang *et al.*, 2009*b*), as similar as the value of the similar bond distance of 1.503 (2)Å (Feng *et al.*, 2010).

Classical hydrogen bonds (O—H···O and N—H···O) are found in the cystal structure (Fig. 2 & Table 1) are essential forces in crystal formation.

### Experimental

Racemic chlorprenaline was prepared by chlorprenaline hydrochloride purchased from ShangHai Shengxin Medicine & Chemical Co., Ltd. ShangHai, China. chlorprenaline hydrochloride and NaOH in a molar ratio of 1:1 were mixed and dissolved in a methanol–water solution (1:1 *v*/*v*). The precipitate formed was filtered off, washed with water and dried. It was used without further purification. Racemic chlorprenaline (0.5 g, 0.0023 mol) was dissolved in methanol (5 ml) and then Benzoic acid (0.3 g, 0.0023 mol) was added. The mixture was dissovled by heating to 323 K where a clear solution resulted. The resulting solution was concentrated at ambient temperature. Colourless crystals of I separated from the solution in about 68% yield after one day.

### Refinement

All of the H atoms were placed in calculated positions and allowed to ride on their parent atoms at distances of 0.93Å (aromatic), 0.98Å (methine), 0.97Å (methylene), 0.96Å (methyl) 0.82Å (hydroxyl) and N—H = 0.90Å, with *U*_iso_(H) = 1.2–1.5 *U*_eq_(C,O,N).

### Crystal data

**Table:**

C_11_H_17_ClNO^+^·C_7_H_5_O_2_^−^	*F*(000) = 712
*M**_r_* = 335.82	*D*_x_ = 1.227 Mg m^−^^3^
Monoclinic, *P*2_1_/*n*	Mo *K*α radiation, λ = 0.71073 Å
Hall symbol: -P 2yn	Cell parameters from 12983 reflections
*a* = 7.8343 (3) Å	θ = 3.0–27.4°
*b* = 13.1260 (5) Å	µ = 0.22 mm^−^^1^
*c* = 17.7308 (7) Å	*T* = 296 K
β = 94.330 (1)°	Chunk, colourless
*V* = 1818.11 (12) Å^3^	0.53 × 0.48 × 0.46 mm
*Z* = 4	

### Data collection

**Table:**

Rigaku R-AXIS RAPID/ZJUG diffractometer	4123 independent reflections
Radiation source: rolling anode	3186 reflections with *I* > 2σ(*I*)
graphite	*R*_int_ = 0.023
Detector resolution: 10.00 pixels mm^-1^	θ_max_ = 27.4°, θ_min_ = 3.0°
ω scans	*h* = −9→10
Absorption correction: multi-scan (*ABSCOR*; Higashi, 1995)	*k* = −15→17
*T*_min_ = 0.871, *T*_max_ = 0.904	*l* = −22→22
17400 measured reflections	

### Refinement

**Table:**

Refinement on *F*^2^	Secondary atom site location: difference Fourier map
Least-squares matrix: full	Hydrogen site location: inferred from neighbouring sites
*R*[*F*^2^ > 2σ(*F*^2^)] = 0.042	H-atom parameters constrained
*wR*(*F*^2^) = 0.110	*w* = 1/[σ^2^(*F*_o_^2^) + (0.0437*P*)^2^ + 0.6461*P*] where *P* = (*F*_o_^2^ + 2*F*_c_^2^)/3
*S* = 1.00	(Δ/σ)_max_ < 0.001
4123 reflections	Δρ_max_ = 0.31 e Å^−^^3^
210 parameters	Δρ_min_ = −0.32 e Å^−^^3^
0 restraints	Extinction correction: *SHELXL97* (Sheldrick, 2008), Fc^*^=kFc[1+0.001xFc^2^λ^3^/sin(2θ)]^-1/4^
Primary atom site location: structure-invariant direct methods	Extinction coefficient: 0.0125 (12)

### Special details

**Table:**

Geometry. All s.u.'s (except the s.u. in the dihedral angle between two l.s. planes) are estimated using the full covariance matrix. The cell s.u.'s are taken into account individually in the estimation of s.u.'s in distances, angles and torsion angles; correlations between s.u.'s in cell parameters are only used when they are defined by crystal symmetry. An approximate (isotropic) treatment of cell s.u.'s is used for estimating s.u.'s involving l.s. planes.
Refinement. Refinement of *F*^2^ against ALL reflections. The weighted *R*-factor *wR* and goodness of fit *S* are based on *F*^2^, conventional *R*-factors *R* are based on *F*, with *F* set to zero for negative *F*^2^. The threshold expression of *F*^2^ > σ(*F*^2^) is used only for calculating *R*-factors(gt) *etc*. and is not relevant to the choice of reflections for refinement. *R*-factors based on *F*^2^ are statistically about twice as large as those based on *F*, and *R*-factors based on ALL data will be even larger.

### Fractional atomic coordinates and isotropic or equivalent isotropic displacement parameters (Å^2^)

**Table:**

	*x*	*y*	*z*	*U*_iso_*/*U*_eq_	
Cl1	0.45832 (8)	0.73075 (5)	0.69565 (4)	0.0898 (2)	
N1	0.27625 (14)	0.59370 (8)	0.48693 (6)	0.0326 (2)	
H1A	0.2765	0.5272	0.4744	0.039*	
H1B	0.3858	0.6149	0.4912	0.039*	
O3	0.40196 (12)	0.31773 (8)	0.50725 (6)	0.0456 (3)	
O2	0.23389 (16)	0.40526 (8)	0.42406 (7)	0.0543 (3)	
O1	0.25272 (15)	0.43338 (8)	0.61142 (6)	0.0497 (3)	
H1	0.3143	0.4081	0.5810	0.075*	
C13	0.21403 (17)	0.22582 (10)	0.42001 (8)	0.0369 (3)	
C8	0.20481 (18)	0.60417 (11)	0.56199 (8)	0.0392 (3)	
H8A	0.0844	0.5862	0.5571	0.047*	
H8B	0.2135	0.6748	0.5779	0.047*	
C9	0.18157 (18)	0.65197 (11)	0.42338 (9)	0.0418 (3)	
H9	0.0642	0.6258	0.4164	0.050*	
C1	0.3003 (2)	0.65860 (13)	0.73480 (9)	0.0522 (4)	
C14	0.1011 (2)	0.22632 (12)	0.35609 (9)	0.0463 (4)	
H14	0.0760	0.2873	0.3309	0.056*	
C12	0.28937 (17)	0.32356 (10)	0.45198 (8)	0.0373 (3)	
C6	0.23953 (18)	0.57020 (11)	0.69877 (8)	0.0410 (3)	
C7	0.29652 (18)	0.53722 (10)	0.62261 (8)	0.0369 (3)	
H7	0.4206	0.5458	0.6218	0.044*	
C10	0.2707 (2)	0.63197 (14)	0.35160 (9)	0.0545 (4)	
H10A	0.2723	0.5600	0.3419	0.082*	
H10B	0.3860	0.6571	0.3577	0.082*	
H10C	0.2100	0.6661	0.3098	0.082*	
C11	0.1747 (3)	0.76461 (13)	0.44186 (11)	0.0617 (5)	
H11A	0.1177	0.7740	0.4874	0.093*	
H11B	0.1130	0.8000	0.4010	0.093*	
H11C	0.2890	0.7911	0.4488	0.093*	
C16	0.0598 (3)	0.04706 (15)	0.36633 (13)	0.0800 (7)	
H16	0.0070	−0.0128	0.3489	0.096*	
C18	0.2509 (3)	0.13432 (13)	0.45566 (11)	0.0644 (5)	
H18	0.3292	0.1323	0.4978	0.077*	
C15	0.0252 (2)	0.13674 (14)	0.32929 (11)	0.0625 (5)	
H15	−0.0495	0.1377	0.2860	0.075*	
C5	0.1165 (2)	0.51537 (15)	0.73318 (9)	0.0572 (4)	
H5	0.0756	0.4549	0.7113	0.069*	
C3	0.1146 (3)	0.63805 (19)	0.83306 (11)	0.0776 (7)	
H3	0.0712	0.6611	0.8774	0.093*	
C17	0.1724 (4)	0.04543 (14)	0.42930 (14)	0.0901 (8)	
H17	0.1961	−0.0157	0.4545	0.108*	
C2	0.2388 (3)	0.69251 (17)	0.80124 (10)	0.0688 (6)	
H2	0.2816	0.7519	0.8241	0.083*	
C4	0.0528 (3)	0.5488 (2)	0.79991 (11)	0.0783 (6)	
H4	−0.0309	0.5113	0.8221	0.094*	

### Atomic displacement parameters (Å^2^)

**Table:**

	*U*^11^	*U*^22^	*U*^33^	*U*^12^	*U*^13^	*U*^23^
Cl1	0.0863 (4)	0.0823 (4)	0.1027 (4)	−0.0357 (3)	0.0192 (3)	−0.0458 (3)
N1	0.0330 (5)	0.0275 (5)	0.0370 (6)	−0.0016 (4)	0.0007 (4)	−0.0014 (4)
O3	0.0384 (5)	0.0479 (6)	0.0497 (6)	−0.0055 (5)	−0.0019 (4)	−0.0129 (5)
O2	0.0723 (8)	0.0301 (5)	0.0588 (7)	−0.0024 (5)	−0.0053 (6)	−0.0076 (5)
O1	0.0659 (7)	0.0344 (5)	0.0512 (6)	−0.0035 (5)	0.0199 (5)	−0.0064 (5)
C13	0.0375 (7)	0.0323 (7)	0.0412 (7)	−0.0023 (6)	0.0046 (6)	−0.0077 (6)
C8	0.0381 (7)	0.0382 (7)	0.0416 (8)	0.0030 (6)	0.0053 (6)	−0.0045 (6)
C9	0.0370 (7)	0.0397 (8)	0.0473 (8)	−0.0004 (6)	−0.0054 (6)	0.0072 (6)
C1	0.0531 (9)	0.0565 (10)	0.0458 (9)	0.0081 (8)	−0.0039 (7)	−0.0146 (7)
C14	0.0491 (8)	0.0384 (8)	0.0502 (9)	0.0006 (7)	−0.0040 (7)	−0.0073 (6)
C12	0.0355 (7)	0.0351 (7)	0.0419 (7)	−0.0045 (6)	0.0074 (6)	−0.0090 (6)
C6	0.0425 (7)	0.0454 (8)	0.0348 (7)	0.0094 (6)	0.0005 (6)	−0.0031 (6)
C7	0.0381 (7)	0.0342 (7)	0.0386 (7)	0.0006 (6)	0.0050 (6)	−0.0052 (5)
C10	0.0610 (10)	0.0605 (10)	0.0410 (8)	0.0011 (8)	−0.0027 (7)	0.0093 (7)
C11	0.0728 (12)	0.0393 (9)	0.0729 (12)	0.0131 (8)	0.0032 (9)	0.0116 (8)
C16	0.1048 (17)	0.0444 (10)	0.0876 (15)	−0.0270 (11)	−0.0145 (13)	−0.0166 (10)
C18	0.0916 (14)	0.0393 (8)	0.0581 (11)	−0.0045 (9)	−0.0216 (10)	−0.0008 (8)
C15	0.0630 (11)	0.0572 (11)	0.0642 (11)	−0.0086 (9)	−0.0147 (9)	−0.0186 (9)
C5	0.0658 (11)	0.0623 (11)	0.0453 (9)	0.0028 (9)	0.0154 (8)	0.0000 (8)
C3	0.0973 (16)	0.1001 (17)	0.0361 (9)	0.0424 (14)	0.0090 (10)	−0.0085 (10)
C17	0.144 (2)	0.0329 (9)	0.0878 (16)	−0.0147 (11)	−0.0269 (15)	0.0046 (9)
C2	0.0803 (13)	0.0757 (13)	0.0483 (10)	0.0247 (11)	−0.0089 (9)	−0.0237 (9)
C4	0.0884 (15)	0.0955 (17)	0.0549 (11)	0.0159 (13)	0.0311 (11)	0.0092 (11)

### Geometric parameters (Å, °)

**Table:**

Cl1—C1	1.7438 (19)	C6—C7	1.5169 (19)
N1—C8	1.4884 (17)	C7—H7	0.9800
N1—C9	1.5096 (17)	C10—H10A	0.9600
N1—H1A	0.9000	C10—H10B	0.9600
N1—H1B	0.9000	C10—H10C	0.9600
O3—C12	1.2704 (17)	C11—H11A	0.9600
O2—C12	1.2456 (18)	C11—H11B	0.9600
O1—C7	1.4158 (16)	C11—H11C	0.9600
O1—H1	0.8200	C16—C15	1.365 (3)
C13—C18	1.378 (2)	C16—C17	1.370 (3)
C13—C14	1.384 (2)	C16—H16	0.9300
C13—C12	1.5050 (18)	C18—C17	1.384 (3)
C8—C7	1.526 (2)	C18—H18	0.9300
C8—H8A	0.9700	C15—H15	0.9300
C8—H8B	0.9700	C5—C4	1.390 (3)
C9—C11	1.516 (2)	C5—H5	0.9300
C9—C10	1.520 (2)	C3—C2	1.363 (3)
C9—H9	0.9800	C3—C4	1.382 (3)
C1—C2	1.380 (2)	C3—H3	0.9300
C1—C6	1.391 (2)	C17—H17	0.9300
C14—C15	1.386 (2)	C2—H2	0.9300
C14—H14	0.9300	C4—H4	0.9300
C6—C5	1.381 (2)		
			
C8—N1—C9	115.08 (11)	C6—C7—H7	109.8
C8—N1—H1A	108.5	C8—C7—H7	109.8
C9—N1—H1A	108.5	C9—C10—H10A	109.5
C8—N1—H1B	108.5	C9—C10—H10B	109.5
C9—N1—H1B	108.5	H10A—C10—H10B	109.5
H1A—N1—H1B	107.5	C9—C10—H10C	109.5
C7—O1—H1	109.5	H10A—C10—H10C	109.5
C18—C13—C14	118.61 (14)	H10B—C10—H10C	109.5
C18—C13—C12	120.50 (13)	C9—C11—H11A	109.5
C14—C13—C12	120.84 (13)	C9—C11—H11B	109.5
N1—C8—C7	112.82 (11)	H11A—C11—H11B	109.5
N1—C8—H8A	109.0	C9—C11—H11C	109.5
C7—C8—H8A	109.0	H11A—C11—H11C	109.5
N1—C8—H8B	109.0	H11B—C11—H11C	109.5
C7—C8—H8B	109.0	C15—C16—C17	119.85 (16)
H8A—C8—H8B	107.8	C15—C16—H16	120.1
N1—C9—C11	110.87 (13)	C17—C16—H16	120.1
N1—C9—C10	107.86 (12)	C13—C18—C17	120.49 (17)
C11—C9—C10	112.06 (14)	C13—C18—H18	119.8
N1—C9—H9	108.7	C17—C18—H18	119.8
C11—C9—H9	108.7	C16—C15—C14	120.15 (17)
C10—C9—H9	108.7	C16—C15—H15	119.9
C2—C1—C6	122.12 (18)	C14—C15—H15	119.9
C2—C1—Cl1	117.95 (15)	C6—C5—C4	121.29 (19)
C6—C1—Cl1	119.92 (12)	C6—C5—H5	119.4
C13—C14—C15	120.55 (15)	C4—C5—H5	119.4
C13—C14—H14	119.7	C2—C3—C4	120.44 (18)
C15—C14—H14	119.7	C2—C3—H3	119.8
O2—C12—O3	124.03 (13)	C4—C3—H3	119.8
O2—C12—C13	117.97 (12)	C16—C17—C18	120.32 (19)
O3—C12—C13	117.97 (13)	C16—C17—H17	119.8
C5—C6—C1	117.24 (15)	C18—C17—H17	119.8
C5—C6—C7	120.44 (14)	C3—C2—C1	119.36 (19)
C1—C6—C7	122.23 (14)	C3—C2—H2	120.3
O1—C7—C6	108.36 (12)	C1—C2—H2	120.3
O1—C7—C8	111.07 (12)	C3—C4—C5	119.5 (2)
C6—C7—C8	107.95 (11)	C3—C4—H4	120.2
O1—C7—H7	109.8	C5—C4—H4	120.2
			
C9—N1—C8—C7	175.43 (11)	C1—C6—C7—C8	−76.00 (17)
C8—N1—C9—C11	57.38 (16)	N1—C8—C7—O1	−73.09 (15)
C8—N1—C9—C10	−179.59 (12)	N1—C8—C7—C6	168.25 (11)
C18—C13—C14—C15	0.9 (3)	C14—C13—C18—C17	−2.0 (3)
C12—C13—C14—C15	−176.53 (15)	C12—C13—C18—C17	175.4 (2)
C18—C13—C12—O2	−170.12 (16)	C17—C16—C15—C14	−1.3 (4)
C14—C13—C12—O2	7.3 (2)	C13—C14—C15—C16	0.7 (3)
C18—C13—C12—O3	7.8 (2)	C1—C6—C5—C4	1.8 (3)
C14—C13—C12—O3	−174.79 (13)	C7—C6—C5—C4	−174.81 (17)
C2—C1—C6—C5	−1.5 (2)	C15—C16—C17—C18	0.2 (4)
Cl1—C1—C6—C5	178.90 (13)	C13—C18—C17—C16	1.5 (4)
C2—C1—C6—C7	175.02 (15)	C4—C3—C2—C1	1.1 (3)
Cl1—C1—C6—C7	−4.6 (2)	C6—C1—C2—C3	0.1 (3)
C5—C6—C7—O1	−19.97 (19)	Cl1—C1—C2—C3	179.68 (15)
C1—C6—C7—O1	163.62 (14)	C2—C3—C4—C5	−0.8 (3)
C5—C6—C7—C8	100.41 (16)	C6—C5—C4—C3	−0.7 (3)

### Hydrogen-bond geometry (Å, °)

**Table:**

*D*—H···*A*	*D*—H	H···*A*	*D*···*A*	*D*—H···*A*
N1—H1A···O2	0.90	1.85	2.7231 (15)	162
O1—H1···O3	0.82	1.93	2.7219 (15)	162
N1—H1B···O3^i^	0.90	1.88	2.7710 (15)	169

Symmetry codes: (i) −*x*+1, −*y*+1, −*z*+1.
